# Investigating adherence to Australian nutritional care guidelines in patients with head and neck cancer

**DOI:** 10.1186/s41199-018-0033-9

**Published:** 2018-07-31

**Authors:** Sophie Hofto, Jessica Abbott, James E. Jackson, Elisabeth Isenring

**Affiliations:** 1 Faculty of Health Sciences and Medicine, Bond University, 2 Promethean Way, Robina, QLD 4226 Australia; 2 Gold Coast University Hospital, Southport, QLD Australia; 3 Radiation Oncology Centres, Gold Coast University Hospital, Southport, QLD Australia

**Keywords:** Head and neck cancer, Nutrition, Dietitian, Radiation therapy, Gastrostomy, PG-SGA

## Abstract

**Background:**

Significant weight loss and malnutrition are common in patients with head and neck cancer, despite advances in treatment and development of evidenced-based guidelines. The aim of this study was to assess adherence to evidenced-based guidelines and investigate nutrition outcomes during and post radiation treatment in head and neck cancer patients.

**Methods:**

This was a two-year retrospective cohort study of 209 head and neck cancer patients (85% male) treated with ≥20 fractions of radiation (mean dose = 64.8 Gy delivered over 31.9 fractions) at an Australian tertiary hospital.

**Results:**

Regarding guideline adherences, 80% of patients were seen by a dietitian weekly during treatment and 62% of patients were seen bi-weekly for six-weeks post-treatment. Average weight loss was 6.7% during treatment and 10.3% three-months post treatment. At the end of treatment, oropharyngeal and oral cavity patients had lost the most weight (8.8, 10.9%), with skin cancer and laryngeal patients losing the least weight (4.8, 2.9%). Gastrostomy patients (*n* = 60) had their tube in-situ for an average of 150 days and lost an average of 7.7 kg (9.4%) during treatment and 11.5 kg (13.5%) from baseline to three-months post treatment. The number of malnourished patients increased from 15% at baseline to 56% at the end of treatment, decreasing to 30% three-months post treatment.

**Conclusions:**

Despite high adherence to evidenced-based guidelines, large discrepancies in weight loss and nutritional status between tumor sites was seen. This highlights the opportunity for further investigation of the relationship between tumor site, nutritional status and nutrition interventions, which may then influence future evidenced-based guidelines.

## Background

As the global incidence of head and neck cancer (HNC) increases, there is a corresponding rise in clinician concern for maximising patient nutritional status [1]. The proximity of tumors to structures vital in mastication and deglutition often result in patients being unable to meet their nutrition and hydration requirements orally [2]. Complications of HNC can include dysphagia, odynophagia, impaired saliva production and changes in speech and breathing [3, 4]. Approximately 25–50% of HNC patients have a significant decrease in dietary intake prior to the commencement of anti-cancer therapy, due to the cancer directly affecting function of the upper aero-digestive tract [5]. In addition, most patients lose in excess of 5% body weight prior to the commencement of treatment [6] and are likely to lose at least 10% of their body weight during treatment, contributing to high rates of protein-energy malnutrition (PEM) [5]. PEM is associated with decreased quality of life, decreased effectiveness of treatment (due to treatment disruptions), increased healthcare costs associated with unplanned hospital admissions, and may negatively impact survival in HNC [7, 8].

Dietetic interventions can help HNC patients meet their nutrition requirements while they are experiencing toxicities of radiotherapy or chemo-radiotherapy [9, 10]. Interventions may include high energy-high protein diets, oral nutrition supplementation and enteral tube feeding. There are two main strategies for enteral feeding in HNC patients, a nasogastric tube (NGT) and a gastrostomy tube, both of which have demonstrated effectiveness in achieving optimal nutritional intake in this population [11]. Proactive placement of a gastrostomy tube is seen as the optimal method to reduce unplanned hospital admissions and treatment interruptions [3], with early intervention associated with improved treatment tolerance, nutritional status, and fewer unplanned hospital admissions [2]. To assist clinicians in achieving the best possible outcomes for HNC patients, both the Clinical Oncology Society of Australia (COSA) and the Royal Brisbane Women’s Hospital (RBWH) have developed evidenced-based guidelines [12, 13]. These guidelines include care pathways for HNC patients based on proposed treatments and severity of malnutrition and dysphagia at diagnosis, and provide recommendations for frequency of dietetic care, goals and nutrition interventions.

Despite the introduction of HNC nutritional management guidelines, there is still debate among clinicians as to what the best nutrition interventions for patients are, due to differences in professional judgement and difficulties adopting evidenced-based guidelines into practice [13, 14]. It is also unclear how well institutions are able to implement and follow evidence-based guidelines in clinical practice. The aims of this study were to determine local adherence to the RBWH ‘Swallowing and Nutrition Management Guidelines for Patients with Head and Neck Cancer’ [12] and the COSA ‘Evidence-based practice guidelines for the nutritional management of adult patients with Head and Neck Cancer’ [13], and to determine local nutrition outcomes pre-treatment, at the completion of treatment, one-month post and three-months post treatment.

## Methods

### Study design

Data were retrieved from electronic medical records of HNC patients treated at a single tertiary facility. Participants were eligible for inclusion if they were 18 years or older and received ≥20 fractions of definitive or high dose palliative intent radiation treatment to the head and neck area. Treatment was scheduled on 5 days per week, within a two-year period between the 1st April 2014 and the 31st of March 2016. Ethical clearance was received from the Gold Coast Hospital and Health Service Human Research Ethics and University Committees.

### Standard care

The facility has a HNC multidisciplinary clinic that is attended by surgical specialists, medical and radiation oncologists, cancer care nurses, speech pathologists and dietitians. All patients attending the clinic underwent baseline swallow and communication screening by a speech pathologist, and baseline nutrition risk was assessed by a dietitian using a validated malnutrition risk screening tool. All patients recommended for radiotherapy +/− systemic therapy with any head and neck cancer diagnosis irrespective of subtype are referred to the hospital’s joint radiation oncology speech pathologist and dietitian HNC clinic. Patients having 20 or more fractions of radiotherapy (i.e. 4 weeks or more) are invited to attend the allied health multidisciplinary pre-treatment education session, followed by weekly individual consultations in the joint speech pathologist and dietitian clinic during treatment. Patient’s having shorter courses of radiation (i.e. < 20 fractions) are offered consultation in the clinic, however frequency of consultations is on an individual basis. In the post treatment setting, patients were scheduled for bi-weekly review appointments for the first 6 weeks and could remain in the service for up to 12 months post treatment.

### Measures

Local clinical practice was assessed against four selected recommendations from the COSA guidelines. These were: 1) the use of a validated malnutrition screening tool (MST) [15, 16], 2) nutrition assessment using the Patient-Generated Subjective Global Assessment (PG-SGA), 3) weekly contact with a dietitian during treatment and 4) post treatment dietitian follow up bi-weekly for 6 weeks. Prophylactic gastrostomy placement was assessed against the risk classification of the RBWH guidelines.

Nutritional status was assessed by dietitians using the global rating of the PG-SGA [17], and weight was measured on calibrated digital scales. The PG-SGA is a nutrition assessment tool that was adapted from the Subjective Global Assessment (SGA), specifically for cancer patients. It is based on patients using a check box format to answer questions regarding recent food intake, nutrition impact symptoms, activities and function and short-term weight loss. A physical examination is then performed by a health professional to assess muscle wasting, loss of subcutaneous fat and oedema [18].

Measures were taken at baseline (initial contact with radiation dietitian and speech pathologist), end of treatment, 1 month post treatment (± 1 week) and 3 months post treatment (± 2 weeks). Gastrostomy dependence was defined as patients who relied on their gastrostomy for any nutrition or hydration intake at 6 and 12 months post completion of treatment. At each assessment, clinicians documented a Functional Oral Intake Scale (FOIS) score that was determined from patient reported typical intake prior to attending their appointment. This is a 7-point ordinal scale that quantifies restrictions on oral intake [19, 20].

### Data analysis

All data were de-identified and stored in Microsoft Excel. Statistical analysis of the data was completed using SPSS (version 22.0, 2013, IBM Corp) and Microsoft Excel. Chi-square tests were used to compare categorical variables, and *t* tests were used for continuous variables. Significance was reported at the *P* = < 0.05 level.

## Results

From the 273 patients referred to the joint dietitian and speech pathologist clinic over the two-year period, 231 were eligible for inclusion. A further 22 patients were excluded due to missing data, mainly from patients declining the speech and dietetics service (*n* = 9), failing to attend multiple appointments (resulting in discharge from service as per local guidelines) (*n* = 8) or not completing treatment (*n* = 5). The sample population (*N* = 209) was predominantly male (85%), over the age of 65 years (range 28–93 years). Common cancer primary sites included skin (32%), oropharynx (27%) and oral cavity (22%) (Table 1). The mean dose of radiation treatment received was 64.8 Gy delivered over 31.9 fractions with 92.3% of patients receiving a dose between 60 and 70 Gy. In the 6 months before treatment, a total of 77 (36.8%) patients reported prior weight loss, with 2.9% experiencing > 10% loss of body weight prior to treatment.

**Table 1:** Baseline patient characteristics of head and neck cancer patients treated with radiation therapy at Gold Coast University Hospital between 1st April 2014 and the 31st of March 2016

Patient characteristics	No. of patients [N, (%)]
Gender	
Male	177 (85)
Female	32 (15)
Age (years)	
< 50	26 (12.5)
50–65	86 (41)
> 65	97 (46.5)
Prior weight loss	
0%	133 (64)
< 5%	51 (24)
5- < 10%	19 (9)
> 10%	6 (3)
Site	
Oral cavity	22 (10.5)
Oropharynx	56 (27)
Nasopharynx	8 (4)
Hypopharynx	7 (3)
Larynx	21 (10)
Salivary	19 (9)
Skin primary	67 (32)
Unknown primary	6 (3)
Other	3 (1.5)
T Stage	
T0	24 (11.5)
T1	35 (17)
T2	51 (24.5)
T3	37 (17.5)
T4	48 (23)
Tx	12 (5.5)
Other	2 (1)
N Stage	
0	54 (25.5)
1	35 (17)
2	112 (53.5)
3	5 (2.5)
Unknown	3 (1.5)
M Stage	
0	206 (97.5)
1	3 (1.5)
Treatment	
Radiation therapy	50 (24)
Radiation therapy + chemotherapy	96 (46)
Surgery + radiation therapy	54 (26)
Surgery + radiation therapy and chemotherapy	10 (4)

Regarding adherence to guideline recommendations, use of a validated malnutrition screening tool pre-treatment was met in 86% of cases and the use of a validated nutrition assessment tool during treatment (PG-SGA) was met for 100% of patients. Weekly review by a dietitian during treatment occurred 80% of the time and 62% of patients were reviewed bi-weekly for 6 weeks post treatment. In patients for whom nutrition screening was not completed, this was mainly due to patient non-attendance at the hospital’s multidisciplinary HNC clinic where screening takes place. The main reason (*n* = 37, 90%) patients were not seen weekly during treatment was due to failure to attend scheduled appointments. In the post-treatment setting, the main reasons patients were not seen fortnightly included failure to attend appointments (*n* = 42, 70%) or clinicians deeming a review was not clinically indicated (*n* = 8, 13%). Of the patients who were not seen fortnightly post treatment, 53% had primary tumors of the larynx or skin, who together lost the least amount of weight in the cohort (Table 2).

**Table 2:** Average weight loss and percentage weight loss by tumor subsite at the end of treatment and three-months post treatment

Cancer Site	End of treatment [kg, (SD), (%)]	3 months post treatment [kg, (SD), (%)]
Oral Cavity	7.9 (4.6) (10.1%)	11.7 (5.3) (14.0%)
Oropharynx	7.5 (4.5) (8.9%)	11.0 (5.9) (12.5%)
Hypopharynx	5.9 (5.0) (7.0%)	7.9 (1.7) (10.9%)
Salivary	4.3 (4.1) (5.7%)	7.1 (3.8) (11%)
Nasopharynx	3.6 (3.0) (4.8%)	3.5 (2.0) (4.8%)
Skin	4.1 (3.9) 5.6 (4.8%)	6.0 (6.1) (6.8%)
Larynx	2.6 (3.6) (2.9%)	4.5 (4.9) (4.7%)

Of the 72 patients who were identified as high risk as per the RBWH guidelines, 58 (81%) received a prophylactic gastrostomy. Fourteen patients did not receive a gastrostomy as they declined the procedure (*n* = 5) or the procedure was medically contraindicated and not offered to the patient (*n* = 9). Of these patients, 86% (*n* = 12) were malnourished at the end of treatment, 3 patients accepted reactive nasogastric feeding tubes, and 4 patients had nutrition-related hospital admissions. An additional 2 patients in the overall cohort (*N* = 209) received a reactive gastrostomy (1 oral cavity patient, 1 oropharynx patient) and 8 patients required reactive nasogastric feeding tube placement (4 oropharynx patients, 2 salivary patients, 1 nasopharynx patient, 1 unknown primary).

**Fig. 1:**
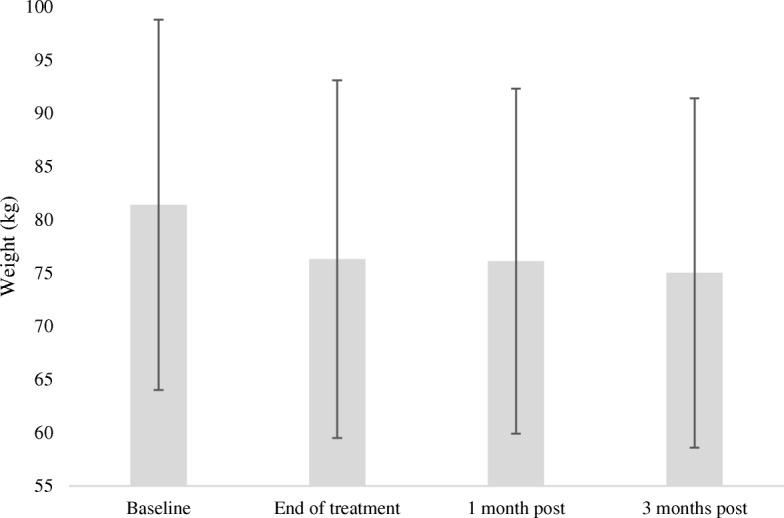
Average weight change for HNC patients from baseline to three months post treatment. Relationship between time and weight loss over a period of three months in head and neck cancer patients over the duration of radiation treatment. Weight change was measure in kilos (kg), with large variations seen at each time point

The average weight loss from baseline at the end of treatment was 5.6 kg (SD 4.5) (6.7%) (*p* = < 0.001), 7.9 kg (SD 5.5) (8.6%) at one-month post treatment (*p* = < 0.001) and 8.9 kg (SD 6.4) (10.3%) 3 months post treatment (*p* = < 0.001) (Fig. 1). The average weight loss was greatest for patients with oral cavity and oropharynx cancers both at the end of treatment (10.9, 8.8%), and three-months post treatment (14.7, 12.5%) (Table 2). Weight loss was statistically significant between oropharyngeal and skin primary cancers at all time points (*p* = < 0.001). Additionally, at the end of treatment, malnutrition rates were statistically higher (*p* = < 0.001) in oropharynx patients (*n* = 41, 77%) in comparison to skin primary cancer patients (*n* = 22, 38%).

Weight loss was not significantly different between patients who received Cetuximab (*n* = 32) and patients who received Cisplatin (*n* = 64) both at the end of treatment (*p* = 0.210) and three-months post treatment (*p* = 0.954). The average weight loss for Cetuximab patients at the end of treatment was 7.3 kg (SD 5.0) (8.9%) and 9.7 kg (SD 6.0) (11.4%) three-months post treatment, compared to 7.4 kg (SD 5.1) (8.6%), and 10.7 kg (SD 6.1) (12.1%) three-months post treatment for Cisplatin patients. A total of 18 (28%) patients who received Cisplatin required a change to Cetuximab during treatment due to toxicity and 23 (36%) of Cisplatin patients planned for 3 high dose cycles either had their dose reduced or third cycle cancelled.

As seen in Table 3, malnutrition rates increased throughout treatment. There was a statistically significant difference in the number of patients malnourished at baseline and the number of patients malnourished at the end of treatment (*p* = < 0.001), the number of patients malnourished one-month post treatment (*p* = < 0.001) and the number of patients malnourished 3 months’ post treatment (*p* = 0.006).

**Table 3:** Patient Generated-Subjective Global Assessment (PG-SGA) global ratings of head and neck cancer patients from baseline to three-months post treatment

Time point	Well-nourished (PG-SGA A) [N, (%)]	Malnourished (PG-SGA B + C) [N, (%)]
Baseline (*n* = 209)	177 (85)	32 (15)
End of Treatment (*n* = 183)	80 (44)	103 (56)
One month post treatment (*n* = 144)	66 (46)	79 (54)
Three months post treatment (*n* = 124)	87 (70)	37 (30)

Changes in type of oral intake as reported by patients and classified by clinicians completing the FOIS were significant between baseline and end of treatment (*p* = < 0.001) and baseline to one-month post treatment (*p* = < 0.001). FOIS scores were not significantly different from baseline to three-months post treatment (*p* = 0.538). The average FOIS score at baseline was 7 (i.e. total oral intake with no restrictions), 4.6 at the end of treatment (i.e. heavily texture-modified diet), 5.6 (texture modified diet) at one–month post treatment and 6 (i.e. near normal diet with special preparation of some foods) three-months post treatment.

Gastrostomy patients (*n* = 60) had their tube in-situ for an average of 150 days (range 44–751 days), with 8 (13%) patients being dependent on their tubes at 6 months post treatment and 2 patients (3%) dependent 12 months post treatment. Gastrostomy patients lost an average of 7.7 kg (9.4%) whilst on treatment and 11.5 kg (13.5%) from baseline to three-months post treatment.

A total of 55 (26%) patients in the cohort had an unplanned hospital admission during treatment, of which 14 (25%) were nutrition-related. The average length of stay was 4.6 days, with common admission reasons including decreased oral intake and anorexia. Of the 60 gastrostomy patients, 24 (40%) required unplanned admissions during treatment, of which 5 (21%) were nutrition-related.

## Discussion

Significant weight loss and malnutrition still occur in HNC patients, despite the introduction of best-practice nutritional guidelines and treatment advances in radiation oncology. Little is known however, how well guidelines are able to be integrated into clinical practice. This study shows that despite good adherence to guidelines selected for comparison, clinically significant weight loss and malnutrition are still highly prevalent. This raises the question as to whether current recommendations for dietetic care both during and post-treatment should be reviewed, particularly for tumor sites which are known to put patients at higher risk for nutritional inadequacy, including those which originate in the oropharynx and oral cavity region.

This study reports an 81% adherence rate to the RBWH HNC risk category guidelines for prophylactic gastrostomy placement, interestingly higher than the 75% reported in 2008 by RBWH [5]. With regards to patient attendance, it was apparent in the current study the main reason patients were not seen as per guideline frequency was due to patient failure to attend. Compliance with appointments in this patient group has previously been reported to be poor [21], with some dietetic clinics stating a non-attendance rate as high as 27% [7]. Non-compliance is more common in patients with mental illnesses, substance abuse, and those who do not feel that dietetic care is a central component of treatment [21].

The average time gastrostomy patients had their feeding tube in-situ (5 months) was substantially shorter than that reported in other studies, with Crombie et al., [22] reporting the average length a patient had their tube in-situ was 7 months and Jack et al., [23] greater than 21 months. This may be due to some services having protocols that recommend gastrostomy tubes remain in for various amounts of time, depending on the treating institutions services and specialist support [13]. Gastrostomy tube dependence is also linked with swallowing outcomes, with literature demonstrating those who are tube-dependent long-term often having minimal swallow function [13]. Stimulation of musculature involved in swallowing has demonstrated to assist patients in returning to a full and non-texture modified diet more quickly post treatment [24]. Although patients in this study showed a decline in their reported ability to consume a full-textured diet during treatment, at three-months post treatment, patient reported dietary restrictions had almost returned to baseline levels in the majority of patients. Decline in the ability to consume a full-textured diet during intensive treatment may reflect a decline in swallow function. Diet restrictions returning to baseline may be in part attributable to the intensive support and prophylactic swallowing exercises patients received as part of the joint dietetic and speech pathologist service.

Weight loss prior to treatment is a prognostic factor for overall survival and is one of the biggest indicators of malnutrition [25]. Malnutrition is strongly related to decreased quality of life, increased unplanned hospital admissions and decreased effectiveness of treatment [7] [8]. The number of patients malnourished both at the end of treatment (56%) and three-months post treatment (30%) is comparable to that demonstrated in the literature. A study by Jager-Wittenaar et al., [26] on oral cavity and oropharyngeal cancer patients, reported that during treatment overall malnutrition rates were 16, and 25% 3 months post treatment [26]. This is similar to van de Berg et al., [27] who conducted a study with squamous cell carcinoma patients, demonstrating even with individual dietary care, 18% of patients were malnourished while receiving treatment and 10.5% were malnourished in the rehabilitation phase two-months post treatment. The differences seen between the current study and both Jager-Wittenaar et al., [26] and van de Berg et al. [27] in malnutrition rates may be due to both the timing of measured outcomes and the difference in definition of malnutrition, with both studies using percentage weight loss to define malnutrition and the current study using the PG-SGA tool.

Clinically significant weight loss (> 10%) prior to treatment was reported by 2.9% of the cohort, less than the 6.6% reported by Brown et al., [5] and the 5% reported by Languis et al., [25]. Weight loss during treatment (average 6.7%) was similar to a study by Langius et al., [25] where patients lost 6.1% body weight during treatment, although greater than that reported by Paccagnella et al., [28] where patients had lost 4.6% of body weight at the end of treatment. The current study also demonstrates that despite continued dietetic intervention, at 3 months post treatment, patients continued to lose weight. This highlights the potential need for increased length of intensive monitoring (bi-weekly) post treatment or additional community support. Weight loss between patients in the current study who received Cisplatin and Cetuximab was not statistically significant both during and post-treatment. It should however be noted, that a large proportion of patients who were planned to receive Cisplatin either did not complete their full course of chemotherapy or changed to Cetuximab to due treatment-related toxicities.

There are currently few studies that demonstrate the difference in weight loss between tumor sites in HNC, despite it being recognised that some tumor sites are at higher risk nutritionally [26]. Weight loss for skin primary HNC patients in particular, is underrepresented in the literature, with some studies not including these tumors in the classification of HNC or grouping these into the ‘other’ category [13, 22, 24, 25, 29]. Consistent with the RBWH guidelines, this demonstrates that those with HNC skin cancer tumors are generally not considered to be at high risk nutritionally. A study by Jager-Wittenaar et al., [29] demonstrated that critical weight loss (> 5% in 1 month or > 10% in 6 months) was seen in 8% of patients with skin, salivary and thyroid tumors, compared to 34% of patients with oropharynx/oral cavity tumors. These results are comparable to those presented in the current study with oropharynx patients losing on average 12.5%, oral cavity patients losing 14.7% and skin cancer patients losing 6.0% of body weight at three-months post treatment. Significant differences (*p* = < 0.001) in weight loss for oral cavity (10%) and oropharynx (11%) patients compared to weight loss in patients with laryngeal tumors (5%) has also been demonstrated in a study by Ehrsson et al., [30]. Likewise, in a study by Nourissat et al., [31] critical weight loss (> 5% during radiation therapy) was reported in 62.9% of oral cavity or oropharynx patients. These studies did not include skin cancer tumors in analysis. Tumor location can then be seen to have a significant impact on both weight loss and nutritional status. As nutritional status exerts its effects not only on the patient, but the healthcare system and dietetic services as well, the relationship between tumor location and weight loss warrants further investigation. Additionally, a review of current guidelines to include more intense nutritional care and updated care pathways for patients at higher risk of clinically significant weight loss (e.g. oropharyngeal and oral cavity tumors) may be justified. Streaming care by tumor site may also reduce unneeded or unnecessary appointments and allow specialised clinicians more time with high risk patients.

The strengths of this study are that the primary researcher who reviewed the outcomes of patients treated at the hospital was not involved in any clinical care of patients, reducing potential researcher bias. The study also had a relatively large sample size and a representative sample of patients recruited. This study is inherently limited by its retrospective, single-institution design. Furthermore, we do not have data on all patients across all the time points, for reasons that include patients transferring back to a local service, thus making it difficult to draw stronger conclusions. A prospective study could overcome this by gathering data of enrolled patients ongoing from outside institutions, should enrolled patients choose to receive follow-up care closer to home. The study is also limited in that it does not address patient adherence to dietary optimization at home or quantify energy and protein intake in comparison to best practice guidelines. Non-adherence to dietetic recommendations may have resulted in higher rates of malnutrition and more significant weight loss. Data on energy and protein intake quantified against guideline recommendations would also demonstrate if patients were meeting their nutrition requirements and still experiencing significant weight loss or malnutrition.

## Conclusion

The use of evidenced-based guidelines for HNC enables the early identification of patients at nutritional risk and provides care pathways for clinicians to follow. Our study supports the use of current Australian guidelines as a method of identifying patients who may need enteral feeding, however suggests that streaming care, in particular for dietetic monitoring by tumor site has the potential to further improve patient outcomes and could be a better use of finite healthcare resources. Continued weight loss in this population post treatment also suggests that perhaps bi-weekly follow up should continue to occur for greater than 6 weeks post treatment. Suggestions for future studies include the trial of care pathways by tumor site to explore weight loss and malnutrition in subgroups of the HNC population, and assessing patient adherence to nutrition recommendations (energy and protein intake) as directed by the dietitian.

## Abbreviations

COSA: Clinical Oncology Society of Australia
FOIS: Functional Oral Intake Scale
HNC: Head and neck cancer
MST: Malnutrition Screening Tool
NGT: Nasogastric Tube
PEG: Percutaneous Gastrostomy Tube
PEM: Protein Energy Malnutrition
PG-SGA: Patient Generated Subjective Global Assessment
RBWH: Royal Brisbane Women’s Hospital.
